# Potential eolian dust contribution to accumulation of selected heavy metals and rare earth elements in the aboveground biomass of *Tamarix* spp. from saline soils in Kazakhstan

**DOI:** 10.1007/s10661-018-7179-0

**Published:** 2019-01-09

**Authors:** Małgorzata Suska-Malawska, Marcin Sulwiński, Mateusz Wilk, Azimbay Otarov, Monika Mętrak

**Affiliations:** 1 Faculty of Biology, Biological and Chemical Research Centre, University of Warsaw, Warsaw, Poland; 2 Kazakh Institute of Soil Science and Agrochemistry, Almaty, Kazakhstan

**Keywords:** Heavy metals, REEs, Soil, Dust pollution, Tamarix spp

## Abstract

In arid and semi-arid zones, atmospheric dust of different origins influences soil chemistry and plant biomass composition. Thus, studies on plant accumulation of heavy metals and rare earth elements (RREs) should include some assessments of potential eolian deposition. Here, we proposed the use of fractionation of metals in soils as an indirect method to assess potential atmospheric dust input to metal content in plant biomass. Our research was performed on individuals of *Tamarix* spp. growing on saline automorphic and hydromorphic soils in Kazakhstan. Studied soils could be, in general, classified as polluted, especially in industrial areas of Karaganda and Chromtau. However, concentrations of heavy metals and RREs in biomass remained low, as most of the studied elements were present in plant-inaccessible forms. Nevertheless, we recorded a high accumulation of Cd in biomass (70% of this element present in soils as plant-inaccessible fractions), which indicates the impact of Cd atmospheric deposition.

## Introduction

Central Asia is a vast area extending from the Caspian Sea to the western borders of China and characterized by highly differentiated landscapes, including high mountains (Tien Shan, Pamir, Altai), excessive desserts, occupying c.a. 60% of this region (Gobi, Karakum, Kyzylkum), and treeless grassy steppes. Developed under arid and semi-arid continental climate, saline soils are dominating in this region, covering over 20% of its desserts and steppes. Automorphic saline soils of Central Asian desserts and semi-deserts are established mostly on parent rocks formed from salt-bearing marine deposits of the Cretaceous period and/or salt-bearing Quaternary sediments (Pankova et al. 2010). Hydromorphic saline soils are azonal and occur mostly in lacustrine and riparian habitats. They are often covered with a salt crust, formed as the result of water level fluctuation and intense evaporation.

Regardless of their origin, saline soils support halophytic vegetation consisting of various salt-tolerant and salt-resisting species. Some of them can grow also on soils contaminated with metals and accumulate their ions (hyperaccumulators). As such, they can be used in the phytoremediation processes (Aralova et al. 2009; Toderich et al. 2010).

Though accumulation and excretion of salts and metals by halophytes were described in details (e.g., Aralova et al. 2009; Sookbirsingh et al. 2010; Flowers and Colmer 2015; Yuan et al. 2016), there are still some problems with the distinction between salts and metals excreted by salt glands (biocrystals) and deposited on plant surface with atmospheric dust (detrital material). In arid and semi-arid zones, atmospheric dust transported at long distances is known to significantly affect soil chemistry (Issanova et al. 2015; Zhu 2016; Opp et al. 2017). Consequently, it will also influence the chemical composition of plant biomass, either remaining in/on the wax layer covering plant surface or entering leaves via stomatal openings. So far, several methods have been suggested to distinguish between plant excretions and dust deposition, including pretreatment methods (washed versus unwashed samples) and measurement methods (Yuan et al. 2016). Yet, they are either questionable or time-consuming and complicated, which makes them unsuitable for monitoring analyses.

Therefore, we suggest using a fractionation of metals in soils (according to a sequential extraction procedure established by the EU Community Bureau of Reference, see Table 1) as an indirect method to assess potential atmospheric dust input to metal content in plant biomass (Pueyo et al. 2008). Assuming that metals present in the soil as fractions bound with sulfates and organic matter and as a residual fraction are plant-inaccessible, their occurrence in plant biomass can be viewed as a result of atmospheric deposition. In the presented research, we studied the abundance of selected metals/metalloids and rare earth elements (REEs) and their fractions in soil and compare it with results obtained for plant biomass to assess the potential influence of atmospheric deposition in the desert regions of Kazakhstan.

**Table 1:** The EU Community Bureau of Reference (CBR) sequential extraction scheme for metals/metalloids and rare earth element (REEs) fractionation in the soil.

Fraction	Phases	Procedure
F1	Mobile/exchangeable elements; bound with carbonates	0.5 g soil with 20 mL 0.1 M NH_4_OAc, shake for 16 h in room temperature
F2	Fe-Mn oxide bound	20 mL 0.1 M NH_2_OH HCl (pH = 2 with HNO_3_), shake for 16 h in room temperature
F3	Sulfide bound and organic matter	30% H_2_O_2_, 5 mL, room temperature, 1 h, followed by 1 h in 85 °C, occasionally shake; add 5 mL 30% H_2_O_2_, 85 °C, 1 h; add 1 M NH_4_OAc (pH 2 with HNO_3_) 25 mL, room temperature, and shake for 16 h
F4	Residues fraction	HNO_3_ + HCl

## Materials and methods

### Study area

In the area of 2.72 Mln km^2^, Kazakhstan is the biggest state in Central Asia. Its landscape is dominated by deserts, semi-deserts, and steppes, covering 80% of the total area. (Pachikin et al. 2014). Our research was conducted in southeastern and southwestern parts of Kazakhstan in two zones representing soil distribution: (1) desert zone with brown and gray-brown soils; (2) dry and desert-steppe zone with chestnut (kastanozems) soils and in the surroundings of rivers Illi and Syr-Darya and Lake Balkhash, where developed azonal hydromorphic soils.

The desert zone with brown and gray-brown soils covers up to 44% of the territory of Kazakhstan, mostly in the southern latitudinal bioclimatic zones, e.g., in undrained lowlands around the Caspian Sea and the Aral Lake, on the Ustyurt Plateau, in the Balkash-Alakol depression, and on sloping foothills of the mountain ranges of Tien-Shan, Zhongariya, Altay, and Saur-Tarbaagatay. Dry and desert-steppe zone with chestnut soils occupies more than 30% of the territory of Kazakhstan and can be characterized by a variety of automorphic soils with alkaline, calcareous, and saline-salt complexes in the soil profiles, which are usually weakly developed. Such soils are typical for the Pre-Ural and Trans-Ural Plateaus, some parts of Caspian lowlands, plain of Irtish, and foothills of Altay and Tarbagatai Mountains (Saparov 2014). Saline marshes develop mostly in riverine deltas, alluvial plains, and lake depressions covered by semi-hydromorphic, hydromorphic, and hypersaline soils (solonchaks) or sandy soils formed by the eolian processes (Pankova et al. 2010).

Soil degradation is a serious problem in Kazakhstan. (Saparov 2014; Pachikin et al. 2014) It is estimated that 75% of the Kazakhstani territory is covered with degraded soils, and 14% of these soils were classified as heavily degraded. According to Laiskhanov et al. 2016), the area of 41% of Kazakhstani soils is saline. Eleven percent out of them are subject to secondary salinization, mostly due to inappropriate irrigation techniques (Otarov 2014). As agriculture plays an important role in the Kazakhstani economy, irrigation-related secondary salinization causes severe losses of arable soils, estimated at several thousands of hectares per year. Apart from industrial pollution, coming mostly from mines and smelters of various metals (including rare earth elements), secondary salinization is the main challenge of the Kazakhstani environmental policy. Currently, pollution and salinity monitoring are one of the top priorities for Kazakhstani research institutions, especially in the context of food security (Kazakh Ministry Report 2018).

### Species used in research

Genus *Tamarix* (tamarisk, salt cedar) belongs to the Tamaricaceae family, which contains 78 species adapted mostly to dry and saline habitats (Christenhusz and Byng 2016). Since species from the *Tamarix* genus excrete excess salts as crystals through glandular cells, they can be classified as recretohalophytes (excretive xerohalophytes) (Albert et al. 2000; Breckle 2002). *Tamarix* species occur in a variety of dry habitats, e.g., on deserts and semi-deserts, saline sandy soils, saline wetlands, and riverine deltas. They are present also in many contaminated sites and were described as accumulators of metals (Manousaki et al. 2008; Toderich et al. 2010; Sookbirsingh et al. 2010; Fawzy et al. 2006). Therefore, *Tamarix* species can be used as bioindicators in the monitoring of soil contamination with metals, especially in arid areas, where high soil salinity limits the number of potential bioindicators.

### Sampling

**Fig. 1:**
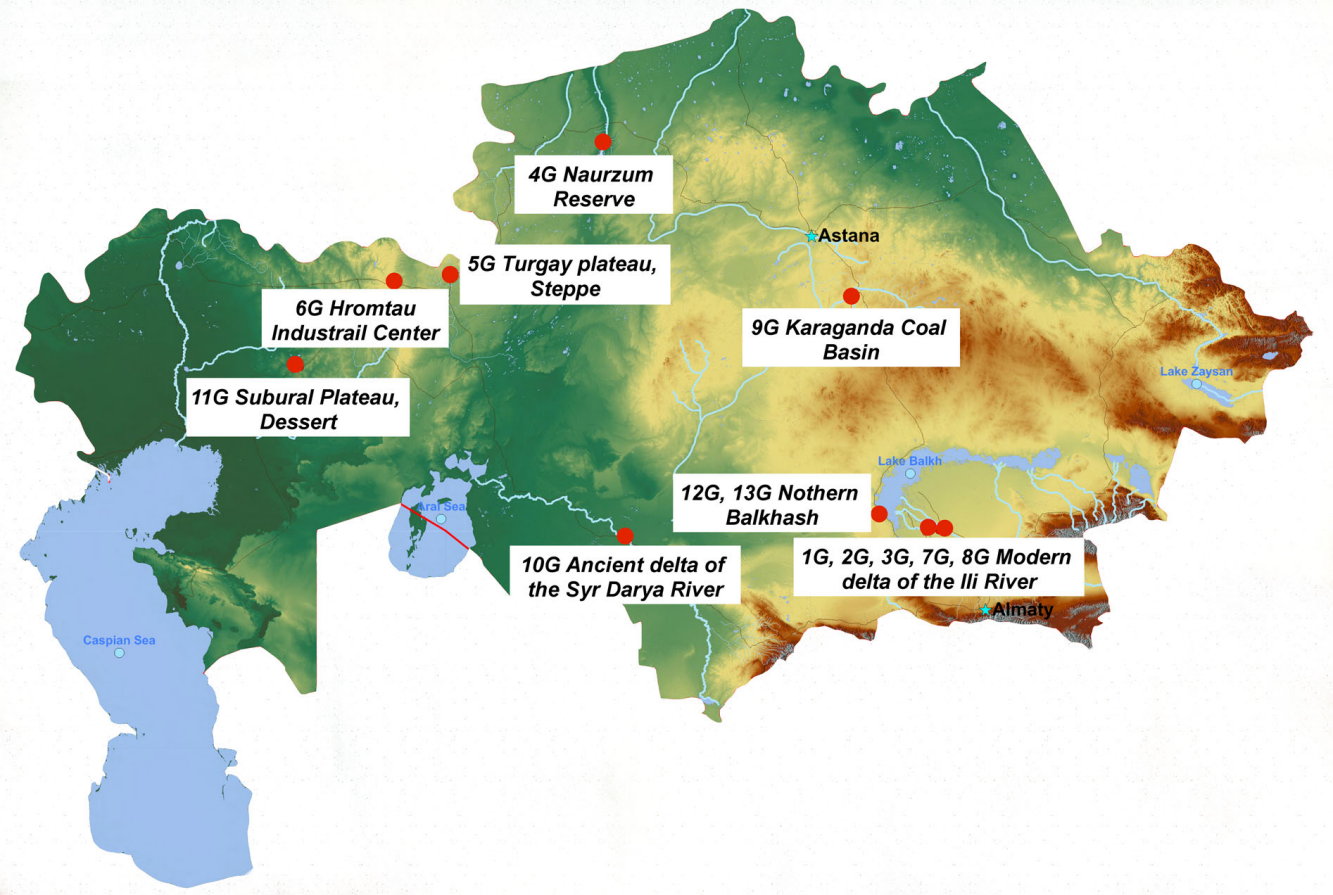
Location of sampling sites in the territory of the Republic of Kazakhstan.

Both soil and plant samples were taken in June 2012 from 13 sites: two sites in the desert zone (9G and 11G), three sites in the desert-steppe zone (4G, 5G, and 6G), six sites in the alluvial plains (1G, 2G, 3G, 7G, 8G, and 10G), and two sites in the lake depression (12G and 13G) (Fig. 1). For the purpose of further analyses, we divided sampled soils into two categories, namely automorphic soils (in our case gray-brown and chestnuts soils developed in desert and steppe zone) and hydromorphic soils (in our case soils formed in river deltas and around lakes).

From each sampling site, we collected a mixed soil sample comprising five subsamples (approximately 50 g in total) from the top 20 cm of the soil in the immediate vicinity of *Tamarix* spp. shrubs. Additionally, a mixed sample of 3–10 branches of an individual *Tamarix spp.* shrub (approximately 20 g of leaves and branches combined) was collected at each sampling site. Soil and plant samples were stored in paper bags and transported to the Laboratory of Biogeochemistry and Environmental Protection at the Biological and Chemical Research Centre of the University of Warsaw.

### Laboratory analyses

Collected soils were air-dried, ground, and sieved through a 1-mm sieve to remove stones, roots, and other large particles. Soil pH was measured in a 1:1 soil/water and 1:1 soil/1M KCl solvents. Soil total carbon (TC), total nitrogen (TN), and total organic carbon (TOC) (after removal carbonates using 10% HCl were determined using CHN Flash 2000 Elementary Analyzer). The soil organic matter (SOM) was calculated as follows SOM = 1.724 TOC (Ruehlmann and Körschens 2009). Electrical conductivity (EC) was measured with Hach HQ40d device in water extract (soil:water ratio 1:5) and recalculated to the electrical conductivity of soil saturated extract (ECe) at 25 °C [dS/m] (Shirokova et al. 2000). For the assessment of total concentrations of metals/metalloids (Be, Cd, Co, Cr, Cs, Cu, La, Mn, Mo, Ni, Pb, Se, Sn, Tl, and Zn) and REEs (Ce, Nd, Pr, and Zr) in soil samples, a three-stage-modified procedure proposed by the EU Community Bureau of Reference was used (Table 1) (Pueyo et al. 2008). The measurements were performed by Inductively Coupled Plasma Mass Spectrometer ICP MS (Perkin Elmer NexION 300D).

For the assessment of total concentrations of metals/metalloids (Be, Cd, Co, Cr, Cs, Cu, La, Mn, Mo, Ni, Pb, Se, Sn, Tl, and Zn) and REEs (Ce, Nd, Pr, and Zr) in plant samples, plants were air-dried and ground to a fine powder with the centrifugation mill. Subsamples of 0.1 g of plant material were decomposed in 6 mL of 65 % HNO_3_ (Ultranal) in a Berghof SpeedWave 4 microwave digestion system. Concentrations of elements in question were measured with Perkin Elmer NexION 300D inductively coupled plasma mass spectrometer.

All reagents used were of analytical reagent grade or better. The accuracy of the elemental analysis was checked by the determination of metals/metalloids and REEs in the standard reference materials of soil (light sandy soil BCR142R, Sigma Aldrich) and leafs using Polish Virginia Tobacco Leaves IC-INCT (LGC standards).

### Statistical analyses

As the parameters studied failed to meet the assumptions of parametric tests (normal distribution and/or equal variances), non-parametric tests were used (Kruskal–Wallis test for comparisons between soil types and Spearman correlation coefficient to estimate relations between soil and plant chemistry). For a better estimation of links between metals/metalloids and REEs content in soils and in plants, bioaccumulation factor (BF) for each element was calculated as a ratio of the content of a certain element in the plant to its content in soil (US E.P.A 2007). All statistical analyses were carried out in STATISTICA 13.

## Results

### General characteristics of the studied soils

Detailed chemical characteristics of the studied soils are presented in Table 2. In the case of salinity and electrical conductivity, both automorphic and hydromorphic soils were most differentiated. Hydromorphic soils tended to have higher values of these parameters, with salinity between 566 and 8900 mg/L and electrical conductivity between 8.47 and 272.66 dS/m. For hydromorphic soils, the highest values of salinity and ECe were recorded in samples from the valley of the Illi River. In comparison, the salinity range of automorphic soils was between 63 and 3640 mg/L and electrical conductivity between 1.92–432 dS/m. However, the observed differences between soil types were not statistically significant. In case of automorphic soils, the highest values of salinity-related parameters were recorded in soils sampled from the industrial areas of Karaganda (3640 mg/L and 432.02 dS/m) and Chromtau Industrial Centre (2130 mg/L and 30.19 dS/m). A pH of the studied soils covered very similar ranges—for hydromorphic soils 7.02 to 7.39 and for automorphic soils 7.01 to 7.30.

**Table 2:** Chemical characteristics of the studied soils.

ID sample	Localization	Soil type WRG		pH (H2O)	pH (KCl)	Salinity [mg/L]	EC [ds/dm]	Total nitrogen [%]	Total carbon [%]	SOM [%]
1G	Illi river modern delta/alluvial plain	Sandy soil	Pasture	8.02	7.17	174	3.1	0.15	3.95	2.47
2G	Illi river modern delta/alluvial plain	Solonchak	Natural grassland	7.92	7.39	566	8.47	0.03	0.89	4.33
3G	Illi river modern delta/alluvial plain	Sandy soil	Natural grassland	7.63	7.18	139	2.12	0.02	0.77	0.77
7G	Illi river modern delta/marsh	Solonchak	Pasture	7.89	7.24	8900	115.2	0.28	3.15	5.37
8G	Illi river modern delta/alluvial plain	Sandy soil	Pasture	7.51	7.22	63	2.22	0.02	0.95	0.54
12G	Balkash lake/depression	Solonchak	Natural grassland	7.9	7.02	586	86.98	0.06	1.43	1.21
13G	Balkash lake/depression	Solonchak	Natural grassland	7.98	7.05	1430	20.83	0.04	1.29	0.75
10G	Syr Darya River ancient delta	Takyrs	Pasture	7.47	7.26	1890	272.66	0.13	1.6	1.91
9G	Karaganda coal basin/semi-desert	Gray-brown	Industry	7.77	7.3	3640	432.02	0.11	2.26	3.9
4G	Naurzum reserve/steppe	Gray-brown	Natural grassland	8.28	7.02	125	1.92	0.12	1.44	2.39
5G	Turgay plateau/steppe	Chesnuts/kastanozems	Industry	8.08	7.26	290	4.42	0.22	4.41	4.78
6G	Hromtau industrial center/steppe	Chesnuts/kastanozems	Industry	8.17	7.08	2130	30.19	0.62	5.82	8.26
11G	Sub-Ural plateau/desert	Chesnuts/kastanozems	Natural grassland	8.08	7.01	876	12.97	0.07	2.26	1.21

Hydromorphic soils contained on average lower amounts of soil organic matter, namely between 0.75 and 5.37% than automorphic soils (from 0.54 to 8.26%). The same trends could be observed for other parameters related to organic matter (TC and TN), yet again, the observed differences had no statistical significance.

Similarly to salinity-related parameters, the content of metals/metalloids and REEs in soil samples was very differentiated and corresponded with sampled location, i.e., soils from industrial areas and from floodplains of the Illi river contained higher levels of these elements than the other samples (Table 3). The content of several elements (Cd, Co, Cr, Cu, Se, and Zn) in soils was significantly correlated with the amount of total organic carbon with *r*^2^ values between 0.60 and 0.69 and *p* < 0.05.

**Table 3:** Total content (mg/kg d.m) of metals/metalloids and REEs in soils and in corresponding plant materials

ID sample	Localization	Soil/plant	Se	Cr	Mn	Co	Cu	Zn	Mo	Cd	Tl	Pb	Ni	Sn	Be	Cs	La	Ce	Pr	Nd	Zr
1G	Illi river modern delta/alluvial plain	Soil	1.77	38.72	9.79	10.89	33.85	163.82	1.59	1.40	0.23	50.28	60.39	6.00	1.83	0.25	12.03	32.61	3.03	12.22	302.05
		Plant	1.47	1.45	17.12	0.22	13.12	59.39	0.54	11.34	n.d.	4.26	1.88	0.71	n.d.	n.d.	0.05	0.07	0.01	0.06	n.d.
2G	Illi river modern delta/alluvial plain	Soil	0.34	21.96	364.55	5.68	18.44	84.09	7.92	0.17	0.28	17.06	31.71	6.79	1.76	0.24	1.63	6.25	0.38	1.46	216.04
		Plant	2.90	0.64	34.61	0.58	2.72	4.50	7.65	0.32	n.d.	0.09	n.d.	0.06	n.d.	n.d.	0.18	0.31	0.04	0.19	n.d.
3G	Illi river modern delta/alluvial plain	Soil	0.40	20.64	226.35	3.87	12.36	54.97	1.95	0.03	0.35	107.17	36.31	8.51	1.89	0.24	5.09	14.54	1.14	4.19	123.87
		Plant	0.73	0.65	60.58	0.21	14.07	39.24	3.08	2.05	n.d.	2.09	n.d.	1.14	n.d.	n.d.	0.29	0.39	0.05	0.17	0.24
7G	Illi river modern delta/marsh	Soil	0.86	33.48	363.81	7.22	45.36	198.29	45.64	0.39	0.30	37.82	46.05	19.17	1.91	0.12	6.09	22.91	1.95	8.18	216.47
		Plant	2.78	0.91	27.22	0.28	10.59	67.57	1.10	11.34	n.d.	3.13	1.75	0.54	n.d.	0.01	0.07	0.13	0.02	0.08	0.18
8G	Illi river modern delta/alluvial plain	Soil	1.56	19.64	246.10	4.09	25.45	59.59	0.82	0.68	0.24	27.46	29.03	3.55	1.19	0.13	4.47	14.17	1.24	5.25	147.77
		Plant	0.42	0.04	126.54	0.05	4.60	12.69	1.10	1.13	n.d.	0.27	n.d.	0.16	n.d.	n.d.	0.07	0.13	0.01	0.08	n.d.
12G	Balkash lake/depression	Soil	0.74	15.85	310.46	6.26	20.96	53.58	2.45	0.06	0.14	43.01	53.83	9.58	0.71	0.21	3.04	6.54	0.71	2.65	117.35
		Plant	1.26	1.20	38.50	0.17	9.21	25.65	0.58	2.46	n.d.	1.21	n.d.	0.24	n.d.	0.05	0.12	0.34	0.03	0.11	n.d.
13G	Balkash lake/depression	Soil	0.37	27.08	392.31	7.23	23.41	65.42	1.26	0.11	0.19	16.53	37.34	3.28	1.15	0.09	3.37	37.65	0.90	3.35	98.25
		Plant	2.33	1.20	21.86	0.15	5.44	23.97	0.86	0.47	n.d.	0.54	n.d.	0.09	n.d.	n.d.	0.04	0.04	0.01	0.05	n.d.
9G	Karaganda coal basin/semi-desert	Soil	1.01	20.57	310.42	5.39	31.84	99.18	1.42	0.41	n.d.	46.83	30.85	8.69	1.01	0.10	2.2	5.52	0.60	2.42	154.37
		Plant	2.30	1.11	42.00	0.32	4.07	40.64	0.44	5.64	n.d.	2.05	n.d.	0.46	n.d.	n.d.	0.20	0.42	0.05	0.20	0.27
10G	Syr Darya river ancient delta	Soil	0.40	18.46	113.14	2.74	10.75	37.65	1.47	0.07	n.d.	6.41	18.50	1.70	0.53	0.05	2.23	11.65	0.66	2.58	49.87
		Plant	0.66	2.84	33.55	0.38	4.37	22.61	0.15	0.69	n.d.	0.19	n.d.	0.11	n.d.	n.d.	0.08	0.16	0.02	0.11	n.d.
4G	Naurzum reserve/steppe	Soil	0.29	11.95	161.81	3.01	12.32	33.38	0.60	0.05	0.21	26.32	20.15	3.29	1.51	0.15	1.61	5.07	0.38	1.42	128.09
		Plant	2.30	1.11	42.00	0.32	4.07	40.64	0.44	5.64	n.d.	2.045	n.d.	0.46	n.d.	n.d.	0.20	0.42	0.05	0.20	0.27
5G	Turgay plateau/steppe	Soil	2.15	83.69	9.78	12.31	40.26	150.71	2.22	1.19	0.05	50.93	70.97	8.61	1.49	0.21	11.33	29.17	2.92	10.87	275.29
		Plant	4.13	2.13	55.26	0.45	8.29	61.09	1.47	0.62	n.d.	0.43	1.23	0.57	n.d.	n.d.	0.04	0.04	0.01	0.03	n.d.
6G	Hromtau Industrial Center/steppe	Soil	3.47	323.51	253.56	41.40	25.74	121.87	3.41	0.41	0.09	34.65	662.44	5.67	3.51	0.19	4.44	14.64	1.23	5.24	15.71
		Plant	0.84	1.55	28.92	0.715	13.47	24.17	0.65	1.28	n.d.	0.7	2.3	0.68	n.d.	n.d.	0.04	0.04	0.01	0.04	n.d.
11G	Sub-Ural Plateau/ desert	Soil	3.61	42.85	411.06	12.46	23.48	118.92	5.18	0.61	0.1	34.15	56.99	23.19	2.44	0.21	3.55	12.72	0.88	3.58	658.20
		Plant	6.31	1.96	92.55	2.57	9.83	52.31	0.53	0.92	n.d.	0.40	2.45	0.27	n.d.	n.d.	0.10	0.32	0.05	0.22	n.d.

*n.d.*, not detected

### Relationship between total content of the studied elements in soils and in plant material

**Fig. 2:**
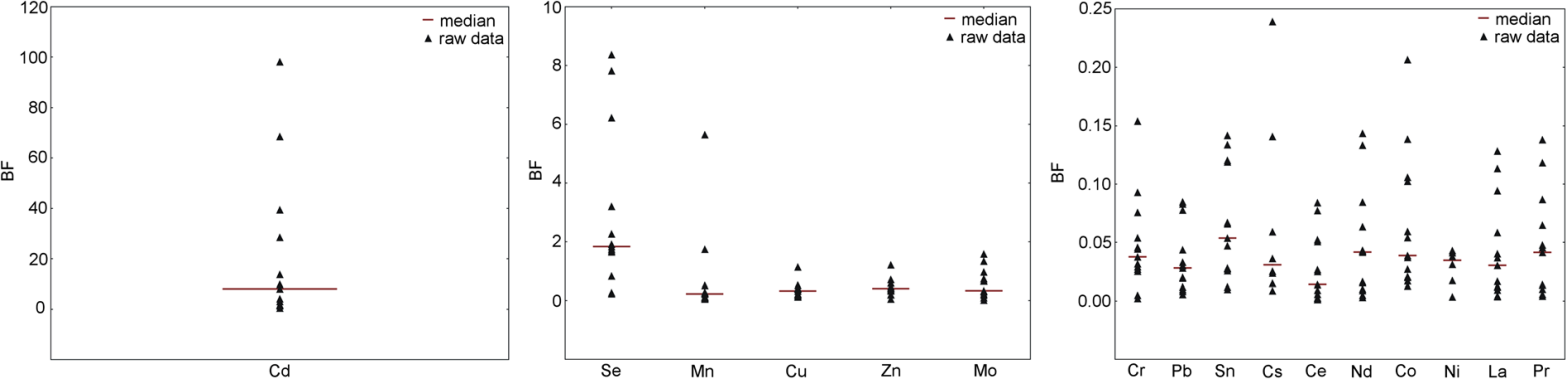
Bioaccumulation factors (BFs) for the studied metals/metalloids and REEs in plant material (*Tamarix* spp.). Due to great differences in BF values, different ranges of Y-axis were used

As in soils, the content of metals/metalloids and REEs in plant material was also very differentiated and usually lower than in corresponding soil samples. Positive correlations between content of metal/metalloid or REEs in soil and in corresponding plant sample were recorded for Zn (*r*^2^ = 0.58, *p* = 0.0375), Pb (r^2^ = 0.63, *p* = 0.0203), and Ni (*r*^2^ = 0.78, *p* = 0.0017). For better assessment of this relation, BF factors were calculated (Fig. 2).

The highest BF values were recorded for Cd and covered a range of 0.52 to 98.17, with a median value of 8.08.

BF values for Cu, Mn, Mo, and Zn were comparable and covered a range between 0.02 and 1.60 (with one higher value for Mn), with a median value between 0.22 and 0.40. The median value for Se was similar (1.84), yet BF values were scattered from 0.24 to 8.36. BFs of other metals and REEs were in a range between 0.001 (Ce) and 0.239 (Cs) with medians in between 0.014 (Ce) and 0.056 (Be).

### Sequential extraction of metals/metalloids and REEs from soils

**Fig. 3:**
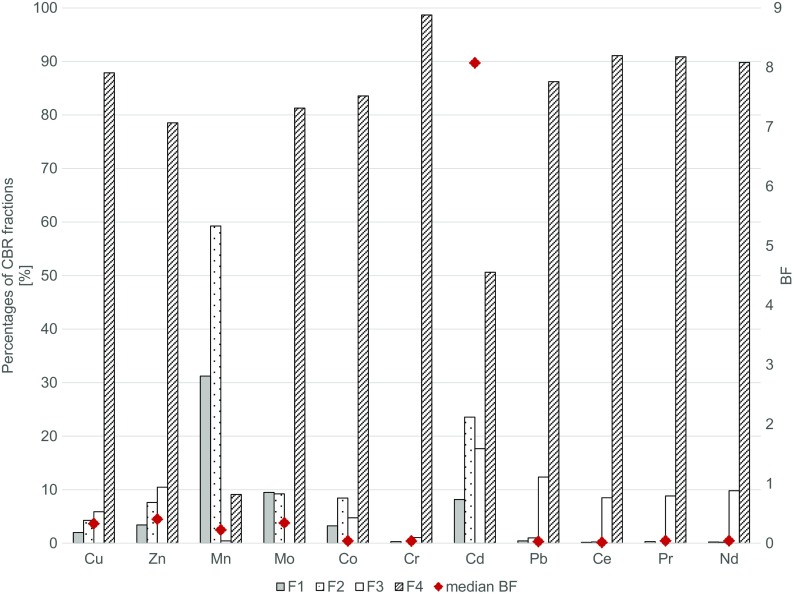
Percentages of different fractions of selected metals and REEs in the studied soils as obtained by the CBR sequential extraction procedure

Percentages of different fractions of selected metals and REEs in the studied soils are presented in Fig. 3.

Most of the studied elements prevail in soils as fractions F4 (alumino-silicates, between 78.5 and 98.7%) and F3 (metalorganic compounds, between 1.0 and 12.3%), with the total amount of F1 and F2 fractions between 0.3 to 6.2%. However, there are two exceptions of this general rule—Cd with the domination of F4 and F3 fractions (consequently, 50.6 and 17.7%), yet the much higher share of F1 and F2 fractions (31.2% in total), and Mn, characterized by a completely different pattern. In the case of Mn, 90.5% of this metal occurred in the soil as F1 (31.2%) and F2 (59.2%) fractions, with only 9.5% of fractions F3 and F4. Excluding the abovementioned exceptions, in the case of REEs (Ce, Pr, and Nd), the total percentage of F1 and F2 fractions remained under 1%, for toxic metals (Cr and Pb) it remained below 10%, and for microelements (Cu, Zn, Mo, and Co) it was in a range from 6 to 20%. General trends observed showed that BFs are positively correlated with percentages of F1 fraction (*r*^2^ = 0.69, *p* = 0.0192) and negatively with percentages of F4 fraction (*r*^2^ = − 0.62, *p* = 0.0402).

## Discussion

Though the salinity of the soils and their content of metals/metalloids and REEs were very differentiated and covered a wide range of values, some general trends were visible. While automorphic soils, especially arenosols, developed on sandy dunes were the least saline and contained the lowest amounts of metals/metalloids and REEs, hydromorphic soils from river valleys showed the highest values of the measured parameters. The latter type of the soils is formed under the constant influence of either surface or ground waters, which combined with intense evaporation in arid zones, results in enhanced salinization. Moreover, hydromorphic soils are relatively rich in organic matter, which effectively binds metal ions. Recent studies on saline hydro- and automorphic soils in Kazakhstan showed that saline meadow soils contained significantly higher amounts of metals than saline chestnut soils. Thus, highlighting the influence of soil organic matter on metal dynamics in saline soils (Sharipow et al. 2017). According to the research of Pankova and Konyushkova (2013), who studied soil salinization in the context of landscape, pedological, and climatic factors, saline hydromorphic soils will be subject to further salinization due to advancing climate warming (intense evaporation leading to increased salinity of ground waters) and agricultural irrigation (extra influx of salts with waters used for irrigation) (Pankova et al. 2010; Pankova and Konyushkova 2013). Moreover, waters used for irrigation in the Central Asia region are very often enriched in various pollutants such as pesticide remaining from fields or toxic metals from mines and smelters. Our observations confirmed the noticeable influence of industrial activities on metals/metalloids and REEs content in soils. However, the increased values of these elements did not necessarily lead to higher bioaccumulation factors. Presumably, due to the domination of plant-inaccessible fractions F3 and F4 in soils. This is also a probable cause of such a small number of significant correlations between the content of elements in soil and in corresponding plant material. Regarding legal Kazakhstani limits of the most frequently monitored metals, they were exceeded in all our samples in the case of Cu (all results over 3 mg/kg) and Zn (all results over 23 mg/kg). In the case of Cd, one third of our samples exceeded the value of 0.5 mg/kg (only one of these samples was from the industrial area), and in case of Pb, half of our samples exceeded the value of 32 mg/kg (including all samples from industrial areas).

Content and composition of salts and metals/metalloids and REEs in soils of arid regions depends on weathering and pedogenic processes that result from the geological and geomorphological characteristic of a given area and are under influence of hydrological and climatic factors. Currently, a major role is played as well by human influences, namely industrial activities, and, especially in arid regions, irrigated agriculture (Kitamura et al. 2006; Satybaldiyev et al. 2015; Laiskhanov et al. 2016; Hamidov et al. 2016). Apart from these factors, many studies performed in arid zones highlighted the importance of eolian dust in shaping soil chemistry, even at a long distance from its original source (Issanova et al. 2015; Zhang et al. 2016; Opp et al. 2017). Dust transported from more or less natural ecosystems, such as the West and Central Asian deserts are well-mixed, with background values of industry-related contaminants and only minor seasonal variations in its composition (Wu et al. 2009, 2015). However, if eolian dust originates in contaminated areas, it contains significant amounts of inorganic and organic pollutants, which is especially well visible in the areas of the dried-out bed of the Aral Lake (Aral-Kum desert). Dust, sand, and salt storms occurring in natural and anthropogenic deserts of Central Asia transport large amounts of deflated material over long distances, significantly affecting agricultural areas as well as human health (Issanova et al. 2015; Zhang et al. 2016; Opp et al. 2017). As the influence of eolian dust (apart from irrigation procedures) was described in the valley of the Illi River (Sommer et al. 2013; Laiskhanov et al. 2016), we believe that increased values of REEs recorded by us in arenosols from this area might result from atmospheric deposition.

Various *Tamarix* species, known as recretohalophytes (excretive xerohalophytes) (Albert et al. 2000; Breckle 2002), are able to accumulate metals from saline and polluted soils (Manousaki et al. 2008; Matsuo et al. 2013). Toderich et al. (2010) recorded remarkably high values of Fe, Ti, Zn, Cu, Sr, and Co in leaves of *Tamarix hipsida* growing on saline and contaminated soils of the Central Kyzylkum desert (Uzbekistan) and proposed to describe this species as a hyperaccumulator (Toderich et al. 2010). In comparison to results described by Toderich et al. (2010), content of Mn, Cr, Co, Mo, Ni, and Be in *Tamarix* individuals presented in this article was lower, while content of Cu, Zn, Pb, and Cd was higher, with values for Cd exceeding these given by Toderich even by three orders of magnitude.

These dissimilarities probably result from different pollution sources and pollution profiles for the Central Kyzylkum desert and our study areas. In the case of Cd, high content recorded for plant biomass resulted in extremely high values of BF for this element (up to 98). According to Manousaki et al. (2008), *Tamarix smyrnesis* growing on saline soils significantly increases absorption of Cd, and, simultaneously, its excretion via salt glands (a detoxification mechanism) (Manousaki et al. 2008).

As in our case, the content of Cd in soils remained on a reasonably low level (between 0.03 and 1.40 mg/kg) and was uncorrelated with BFs; we believe that the accumulation of Cd in the studied *Tamarix* individuals was enhanced by eolian deposits on the surface of plants.

In contrast to metals and metalloids, bioaccessibility of rare earth elements (RREs, as defined by (Tyler 2004; Dołȩgowska and Migaszewski 2013; Srivastava and Kumar 2017), and their influence on plant physiology remains still insufficiently studied. At the same time, their input into the environment is rapidly growing with the development of new technologies. Scientists have described so far several cases of decreased root growth, impaired seed germination, and damaged chlorophyll particles as potential results of REEs accumulation in plant tissues (Li et al. 1998; Tyler 2004; Loell et al. 2011; Dołȩgowska and Migaszewski 2013; Wiche et al. 2017). In our case, REEs content in soils remained much lower than world averages given by (Kabata-Pendias and Pendias 2001) and over 98% of the extracted REEs was in form of plant-inaccessible F3 and F4 fractions. Median values of BFs obtained for REEs in our study were between 0.01 and 0.04 and were lower than BF values recorded at the level of 0.04–0.09 (Migaszewski and Gałuszka 2015). Therefore, we can state, that in our case bioaccessibility of REEs was low and their potential influence on plant development probably negligible.

## Conclusions

- Soils in the studied regions of Kazakhstan can be classified as polluted with Cu, Zn, Cd, and Pb, yet in most cases, bioaccumulation factors remained low and plant biomass was uncontaminated as most of the studied elements were present in plant-inaccessible forms.
- Even if elements were present in plant-accessible forms, we recorded no correlation with their abundance of plant biomass (Mn).
- High accumulation of Cd in biomass, with approximately 70% of this element present in soils as plant-inaccessible fractions, indicates the impact of Cd atmospheric deposition.
- Fractionation of metals in soils can be used as an indirect method for indication of potential atmospheric deposition.
